# Allogeneic hematopoietic stem cell transplantation improves long-term outcome for relapsed AML patients across all ages: results from two East German Study Group Hematology and Oncology (OSHO) trials

**DOI:** 10.1007/s00277-021-04565-1

**Published:** 2021-07-07

**Authors:** Thomas Heinicke, Rainer Krahl, Christoph Kahl, Michael Cross, Sebastian Scholl, Hans-Heinrich Wolf, Detlev Hähling, Ute Hegenbart, Norma Peter, Antje Schulze, Axel Florschütz, Volker Schmidt, Kolja Reifenrath, Niklas Zojer, Christian Junghanss, Herbert G. Sayer, Georg Maschmeyer, Christian Späth, Andreas Hochhaus, Thomas Fischer, Haifa Kathrin Al-Ali, Dietger Niederwieser

**Affiliations:** 1 Department of Hematology and Oncology, University of Magdeburg, Magdeburg, Germany; 2 University Hospital Leipzig, 04106 Leipzig, Germany; 3 Department of Hematology and Oncology, Hospital Magdeburg, Magdeburg, Germany; 4 Klinik Für Innere Medizin II, Universitätsklinikum Jena, Jena, Germany; 5 Department of Hematology and Oncology, University Hospital, Halle, Germany; 6 Department of Hematology and Oncology, Klinikum Schwerin, Schwerin, Germany; 7 Departement of Internal Medicine V, University of Heidelberg, Heidelberg, Germany; 8 Medizinische Klinik, Carl-Thieme-Klinikum GmbH, Cottbus, Germany; 9 Department of Hematology and Oncology, Helios Klinikum Erfurt, Erfurt, Germany; 10 Klinikum Dessau, Dessau, Germany; 11 Klinikum, Zittau, Germany; 12 Department of Medicine I, Wilhelminen Cancer Research Institute, Wilhelminenhospital, , Vienna, Austria; 13 Hematology, Oncology, Palliative Medicine, University of Rostock, Rostock, Germany; 14 Department of Hematology, Oncology and Palliative Care, Ernst Von Bergmann Hospital, Potsdam, Germany; 15 Hematology and Oncology, University of Greifswald, Greifswald, Germany; 16 Lithuanian University of Health Sciences, Kaunas, Lithuania; 17 Aichi Medical University, Nagakute, Japan

**Keywords:** Relapsed acute myeloid leukemia, Prognostic factors, Allogeneic stem cell transplantation

## Abstract

Relapse of acute leukemia is a frequent complication with uncertain outcome and poorly defined risk factors. From 1621 patients entered into two prospective clinical trials (AML02; n = 740 and AML04; n = 881), 74.2% reached complete remission (CR) 1 after induction(s) and 59 patients after additional induction ± hematopoietic cell transplantation (HCT). Of the non-refractory patients, 48.4% with a median age of 63 (range 17–85) years relapsed. Relapses occurred within 6 months after CR in 46.5%, between 7 and 18 months in 38.7%, and after 18 months in 14.8% of patients. Relapse treatment resulted in CR2 in 39% of patients depending upon age (54.5% of ≤ 60 and 28.6% of > 60 years), duration of CR1, and treatment of relapse. Overall survival (OS) was 10.9 (7.4–16.2) %, but OS after HCT ± intensive chemotherapy (ICT) was 39.3% (31.8–48.6) at 5 years and not different in younger and older patients. Donor lymphocyte infusion ± chemotherapy and ICT alone resulted only in OS of 15.4% and of 5%, respectively. Independent favorable factors for OS were long CR1 duration, and HCT, while non-monosomal disease was beneficial for OS in elderly patients. Leukemia-free survival [LFS; 24.9 (19.5–31.7) % at 10 years] was affected by similar risk factors. In a competing risk model, the relapse incidence at 5 years was 53.5 ± 3.5% and the non-relapse mortality rate 21.7 ± 2.9%. Lower relapse incidence was observed in patents with HCT, long CR1 duration, and female gender. Risk factors for non-relapse mortality were HCT in younger and type of AML in elderly patients. In conclusion, allogeneic HCT ± IC improved the results in relapsed AML in younger and elderly patients. Increasing CR2 rates and HCT frequency will be the challenge for the next years. Relapse of the disease remains the major problem.

**Supplementary Information:**

The online version contains supplementary material available at 10.1007/s00277-021-04565-1.

## Introduction

Relapse is the main cause of treatment failure in patients with acute myeloid leukemia (AML). Induction chemotherapy with one or two cycles of cytarabine in combination with anthracyclines results in complete remission (CR) in 60–80% of younger and in 40–60% of older adults depending on genetic and molecular risk factors [1, 2]. Despite achievement of CR following one or two intensive inductions and 2–3 cycles of consolidations with or without hematopoietic cell transplantation (HCT), relapses occur frequently and remain the major obstacle to cure. Prognostic factors for response and survival in relapsed as opposed to newly diagnosed AML are not well defined and are largely restricted to younger patients. In order to identify risk factors in more detail, we analyzed two prospective OSHO studies involving newly diagnosed AML patients. The characteristics, outcome, and prognostic factors were assessed from a total of 1621 AML patients aged 17–87 years. Risk factors for outcome, which include molecular signatures at diagnosis, are particularly important in elderly patients because of their high relapse risk and poor OS [3–8]. The results are important for counseling patients on HCT timing in CR1 or CR2. In addition, the results obtained from this study are compared to those of related studies in recent decades and strategies for improvement are discussed.

## Patients and methods

### Patients

All patients of the prospective OSHO AML trials (AML02 for patients ≤ 60 years, NCT01414231, and AML04 for patients > 60 years of age, NCT01497002) were included. Patients had given informed consent prior to being included in the clinical trials, both of which were approved by the ethics committee of the University of Leipzig.

AML02 included newly diagnosed, non-promyelocytic AML patients aged ≤ 60 years and was part of the German intergroup trial [9]. The upfront randomized (9:1 assignment) intergroup study compared the OSHO study arm to a common standard arm consisting of a 7 + 3 regimen [10]. A total of 30 (8.6%) patients were randomly assigned to the intergroup arm. Patients in the OSHO arm received idarubicin 12 mg/m^2^ qd on days 1–3 and cytarabine 2 g/m^2^ qd on days 1, 3, 5, and 7. After CR, allogeneic HCT was scheduled for patients with adverse or intermediate risk cytogenetics for whom a matched related or unrelated donor was available. AML04 included newly diagnosed, non-promyelocytic AML patients > 60 years of age similarly randomized (9:1 assignment) to receive cytarabine at 2 g/m^2^ qd on days 1, 3, 5, and 7 in combination with mitoxantrone 10 mg/m^2^ qd on days 1–3 (OSHO arm) or the 7 + 3 regimen (n = 43; 9.6%). Allogeneic related or unrelated HCT following non-myeloablative conditioning was considered after CR [11]. AML type (de novo AML, AML following MDS and t-AML) and cytogenetic risk were defined as previously described [12-15]. Treatment of relapse was performed using intensive chemotherapy where possible, hypomethylating agents (HMA), donor lymphocyte infusions (DLI) in patients relapsing after HCT, or HCT.

### Statistical analysis and definitions

CR, partial remission (PR), incomplete remission (CRi), and relapse were defined as published previously [1]. The main study endpoints were OS and LFS. Patients with CR2/CR2i or reinduction failure were compared using the chi-square test or Fisher’s exact test for categorical variables.

OS was calculated from date of diagnosis or relapse until death of any cause. LFS was measured from achievement of CR/CRi until relapse or death in CR. RFS was defined as time to any recurrence of AML, but death was censored. Non-relapse mortality (NRM) was defined as death without prior relapse. For patients without an event, all survival endpoints were censored at the date of last follow-up. Relapse incidence (RI) and NRM were calculated using cumulative incidence in a competing risk setting.

Univariate analyses were done using log-rank test for OS, LFS, and RFS, while the Gray test was applied for RI and NRM [16]. Factors significant at p < 0.1 in univariate analysis were included in the multivariate models. Multivariate analyses were performed using a logistic regression model for response for CR2, the multivariable Cox regression model including allogeneic HCT in treatment of relapse as a time-dependent variable for OS and LFS, and the Fine and Gray regression method for RI and NRM [17]. To evaluate the effect of allogeneic HCT on RI and NRM, we used a landmark analysis taking into the account the median time interval of allogeneic HCT from CR2 (28 days). Patients without event (relapse or death) in the first 28 days after CR2 remained in the HCT comparison group. The degree of relatedness between linear-related variables was calculated by Pearson correlation. Factors in the multivariate model were sequentially removed in the order of least significance until the final model included only factors showing an effect with p < 0.05. All p values reported are two-sided and all statistical analyses were carried out with R (the R project for statistical computing 3.6.0; packages “survival” and “cmprsk”).

## Results

### Patient characteristics at diagnosis and outcome

**Fig. 1:**
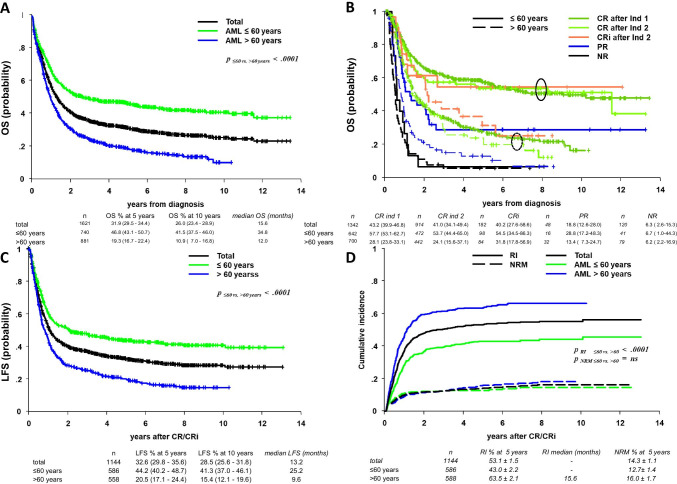
(Newly diagnosed patients). **A** Overall survival for newly diagnosed AML patients (n = 1621), for younger (≤ 60 years; n = 740), and for elderly (> 60 years; n = 881) patients entered in the OSHO studies. **B** Overall survival for all patients, for younger (≤ 60 years), and for elderly (> 60 years) patients (n = 1621) according to remission status. Abbreviations: *CR1 ind.*, after one induction; *CR1 after 2 ind.*, CR1 after two inductions; *PR*, partial remission; *NR*, nonresponse (2 circles in Fig. 1B are showing younger and elderly patients in CR and CRi). **C** Leukemia-free survival (LFS) of patients with AML (all n = 1144) according to age. **D** Non-relapse mortality (NRM) and relapse incidence (RI) for newly diagnosed AML patients according to age (all ages, patients ≤ 60 years and > 60 years)

A total of 1621 newly diagnosed AML patients were recruited. Their median age was 62 (17–87) years, 66.6% had de novo, 25.8% AML following MDS, and 7.6% therapy-related AML (Table 1). The cytogenetic risk was favorable in 7.6%, intermediate in 64.4%, and unfavorable in 27.6% of all patients. An *FLT3-ITD* mutation (mut) was present in 19.1% and *NPM1* mut in 30.2% of patients (with lower frequencies in the elderly patients; p < 0.05). A high proportion of patients (n = 1144; 70.6) reached CR/CRi after one or two courses of induction chemotherapy (ICT) with 238 (14.7%) being considered refractory (56 in the younger and 182 in the elderly population; Table 2). Of these, 59 entered CR after additional ICT or HCT for a total of 1203 patients in CR/CRi (74.2%; Table 1). Only 37.3% of patients received HCT as consolidation in CR1, 46.6% of younger and 27.6% of elderly patients (Table 2). Another 72 of 238 refractory AML patients underwent HCT, corresponding to 69.6% of the younger (≤ 60 years) and 18.1% of the older (> 60 years) patients. OS reached 26.0 (23.4–28.9) % at 10 years with a clear difference between patients ≤ 60 years [41.5 (37.5–46.0) %] and those > 60 years [10.9 (7.0–16.8) %] at 10 years (Fig. 1A; p < 0.0001). OS was dependent upon remission status [CR after one or two induction(s)/CRi vs. PR vs. NR] and age (Fig. 1B). In multivariate analysis, advanced age, cytogenetic risk, and NPM1 *wild type (wt)* were independent risk factors for CR and in addition male gender (p < 0.05), abnormal WBC (< 2 / 2–75 / > 75; p < 0.001), and AML type (p < 0.01) for OS (data not shown). LFS at 10 years was age dependent and amounted to 41.3 (37.0–46.1) % in younger and 15.4 (12.1–19.6) % in elderly patients (Fig. 1C). *FLT3-ITD* was not a risk factor for survival in the whole population, but for RFS in younger patients (p = 0.02; suppl. Figure 1). RI was the predominant complication (53.1 ± 1.5% at 5 years; Fig. 1D) and was clearly higher in elderly (63.5 ± 2.1% at 5 years) than in younger (43.0 ± 2.2% at 5 years) patients. NRM was 14.3 ± 1.1% at 5 years and not age dependent.

**Table 1:** Patient characteristics from diagnosis to relapse

		All patients		≤ 60 years		> 60 years		p-value
		n	%	n	%	n	%	
Total patients, n		1621	100	740	100	881	100	
Age [years]	Median (range)	62 (17–87)		48 (17–60)		69 (60–87)		
AML type, n (%)	De novo Following MDS t-AML Unknown	1075 416 122 8	66.6 25.8 7.6 0.5	571 118 47 4	77.2 16.0 6.3 0.5	504 298 75 4	57.2 33.8 8.5 0.5	< .0001
Cytogenetics, n (%)	Favorable Intermediate Unfavorable Monosomal Unknown	117 947 407 186 150	7.6 64.4 27.6 12.6 9.2	95 404 191 80 50	13.7 58.5 27.6 11.6 6.8	22 543 216 106 100	2.8 69.5 27.6 13.5 11.4	< .0001
Molecular tests, n (%)	*FLT3 ITD* *FLT3 wt* *NPM1 mut* *NPM1 wt*	217 917 339 785	19.1 80.9 30.2 69.8	105 407 171 336	20.5 79.5 33.7 66.3	112 510 168 449	18.0 82.0 27.2 72.8	n.s
								< .05
CR1 after ≤ 2 ICT		1144	70.6	586	79.1	558	63.3	< .0001
CR1 after > 2 ICT or HCT		59	3.6	29	3.9	30	3.4	< .0001
CR/CRi total		1203	74.2	615	83.1	588	66.7	
Relapse		582	48.4	235	40.1	347	62.2	< .0001
Age [years]	Median (range)	63 (17–85)		49 (17–60)		69 (60–85)		
Gender	Male	298	51.2	108	46.0	190	54.8	< .05
AML type	De novo Following MDS Therapy related	389 152 38	67.2 26.3 6.6	187 41 6	79.9 17.5 2.6	202 111 32	58.6 32.2 9.3	< .0001
Cytogenetic risk	Favorable Intermediate Unfavorable Unknown Monosomal	22 347 155 58 76	4.2 66.2 29.6 - 14.5	16 147 58 14 25	7.2 66.5 26.2 - 11.3	6 200 97 44 51	2.0 65.9 32.0 - 16.8	< .01
*FLT3*	wt *ITD*	324 91	78.1 21.9	118 42	73.8 26.3	206 49	80.8 19.2	.09
*NPM1*	wt mut	285 125	69.5 30.5	108 50	68.4 31.6	177 75	70.2 29.8	n.s
CR1–relapse time interval (months)	≤ 6 7–18 ≥ 18	271 225 86	46.6 38.7 14.8	94 99 42	40.0 42.1 17.9	177 126 44	51.0 36.3 12.7	< .05
HCT in CR1	Yes	128	22.0	69	29.4	59	17.0	< .0001
Treatment of relapse	ICT HCT ± prior ICT HMA Mod. CT/palliative DLI ± ICT/mod CT Supportive	190 151 69 65 27 21	36.3 28.9 13.1 12.4 5.2 4.0	71 107 10 9 21 2	32.3 48.6 4.5 4.1 9.5 0.9	119 44 59 56 6 19	39.3 14.5 19.8 18.2 2.0 6.3	< .0001
CR2		227	39.0	128	54.5	99	28.6	< .0001

Abbreviations: *CR*, complete remission; *CRi*, remission with incomplete hematological reconstitution; *FLT3-ITD*, fms-like tyrosine kinase 3–internal tandem duplication; *wt*, wild type; *mut*, mutated; *NPM1*, nucleophosmin-1; *ICT*, induction chemotherapy; *HCT*, hematopoietic cell transplantation; *DLI*, donor lymphocyte infusion; *mod. CT*, low-dose chemotherapy; *HMA*, hypomethylating agents; *n.s.*, not significant

**Table 2:** Frequency of hematopoietic cell transplantation (HCT) in patients with AML

	All patients n (%)			≤ 60 years, n (%)			> 60 years, n (%)		
	1621			740			881		
Disease stage	CR1	Refractory	Relapse/CR2	CR1	Refractory	Relapse/CR2	CR1	Refractory	Relapse/CR2
n	1144	238	582/227	586	56	235/128	558	182	347/99
HCT	427 (37.3)	72 (30.2)	155 (26.6/68.3)	273 (46.6)	39 (69.6)	108 (46.0/84.4)	154 (27.6)	33 (18.1)	47 (13.8/47.4)
Allogeneic HCT	395 (92.5)	72	151 (97.4)	241 (88.3)	39	107 (99.1)	154 (100)	33	44 (93.6)
Related donor	112 (28.4)	20 (27.8)	23 (15.2)	82 (34.0)	12 (30.8)	18 (17.5)^*^	30 (19.5)	8 (24.2)	5 (11.4)^#^
Unrelated donor	282 (71.4)	51 (70.8)	124 (82,1)	158 (66.1)	26 (66.7)	86 (82.6)^*^	124 (80.6)	25 (75.8)	38 (86.4)^#^
MMUD	77 (27.3)	16 (31.4)	41 (33.1)	41 (25.9)	9 (34.6)	36 (42.3)^**^	36 (29.0)	7 (28.0)	5 (13.1)^##^
NMAC	164 (41.5)	14 (19.4)	39 (25.8)	44 (18.3)	5 (12.8)	21 (19.6)	120 (77.9)	9 (27.3)	18 (40.9)
Autologous HCT	32 (7.5)	-	4 (2.6)	32 (11.7)	-	1 (0.9)	-	-	3 (6.4)

Information missing on: ^*^3 donors, ^**^9 × HLA matching, ^#^1 donor, and ^##^1 × HLA matching missing

Abbreviations: *MMUD*, mismatched donor; *NMAC*, non-myeloablative conditioning HCT

### Patient characteristics at relapse and outcome

Of 1148 patients achieving CR/CRi, 48.4% (n = 582) relapsed within 1–121 months (Table 1). Relapse rate was unevenly distributed between patients ≤ 60 and > 60 years with 40.1% and 62.2%, respectively (Table 1; p < 0.0001). Median age at relapse was 63 (range 17 to 85) years as compared to 62 (17–87) years at diagnosis with a slight predominance of male patients (51.2%). While there was a higher number of female patients in the younger population (54%), the elderly cohort contained more males (54.8%; p = 0.04). De novo, AML following MDS and therapy-related AML (*t*-AML) were present in 67.2%, 26.3%, and 6.6% patients, respectively. The frequencies of AML following MDS and of therapy-related AML were higher in elderly than in younger patients (32.2% vs. 17.5% and 9.3% vs. 2.6%, respectively; Table 1; p < 0.0001). Furthermore, 4.2% were of favorable risk, 66.2% of intermediate, and 29.6% of poor risk. Seventy-six patients (14.5%) had a monosomal karyotype at diagnosis (p = 0.007). *FLT3-ITD* was present in 21.9% and *NPM1* mut in 30.5%. Overall, the time interval from CR to relapse was 6 months or less in 46.6%, 7 to 18 months in 38.7%, and 18 months or more in 14.8% patients.

Compared to younger patients, elderly patients had a higher incidence of unfavorable cytogenetics (32.0% vs. 26.2%, respectively; p = 0.007), monosomies (16.8% vs. 11.3%, respectively), and shorter CR1 duration (51.0% vs. 40.0% ≤ 6 months, respectively; p = 0.02; Table 1). There was a tendency towards a lower rate of *FLT3-ITD* in the elderly compared to the younger patients (19.2% vs. 26.3%, respectively; p = 0.09), while *NPM1* mut rates were comparable (29.8 vs. 31.6, respectively; p = n.s.; Table 1).

Treatment of relapse consisted of ICT (n = 190), allogeneic HCT with or without prior ICT (n = 151), palliative low-dose chemotherapy (n = 65), HMA (n = 69), DLI or G-CSF-stimulated buffy coat infusion ± prior chemotherapy (n = 27), best supportive care (n = 21), and tyrosine kinase inhibitors (n = 9). A minority of patients (n = 39) received no treatment for relapse. Numbers of ICT without HCT and, as expected, palliative/supportive treatments were higher in elderly than in younger patients (Table 1). The detailed rate of HCT according to age was 46.0% in younger and 13.8% in elderly patients with relapse (84.3% in younger and 47.7% in elderly patients with CR2) and predominantly from unrelated donors (82.6% and 86.4%, respectively; Table 2). A high proportion of patients was transplanted from mismatched donors (42.3% ≤ 60 years vs. 13.1% > 60 years) and after reduced intensity/non-myeloablative rather than myeloablative conditioning (19.6% ≤ 60 vs. 40.9% > 60 years).

CR2 was attained in 227 (39%) of the 582 patients, with a higher CR rate in patients ≤ 60 (54.5%) than in patients > 60 years (28.6%; Table 1; p < 0.0001). In multivariate analysis, time interval CR–relapse and treatment were independent factors for CR2. Since all other factors correlated with age, younger and elderly patients were analyzed separately (suppl. Table 1). In younger patients, age and monosomal vs. non-monosomal karyotype were additional independent variables, while in elderly patients, only monosomal vs. non-monosomal karyotype and treatment were the only independent variables for achieving CR2.

**Fig. 2:**
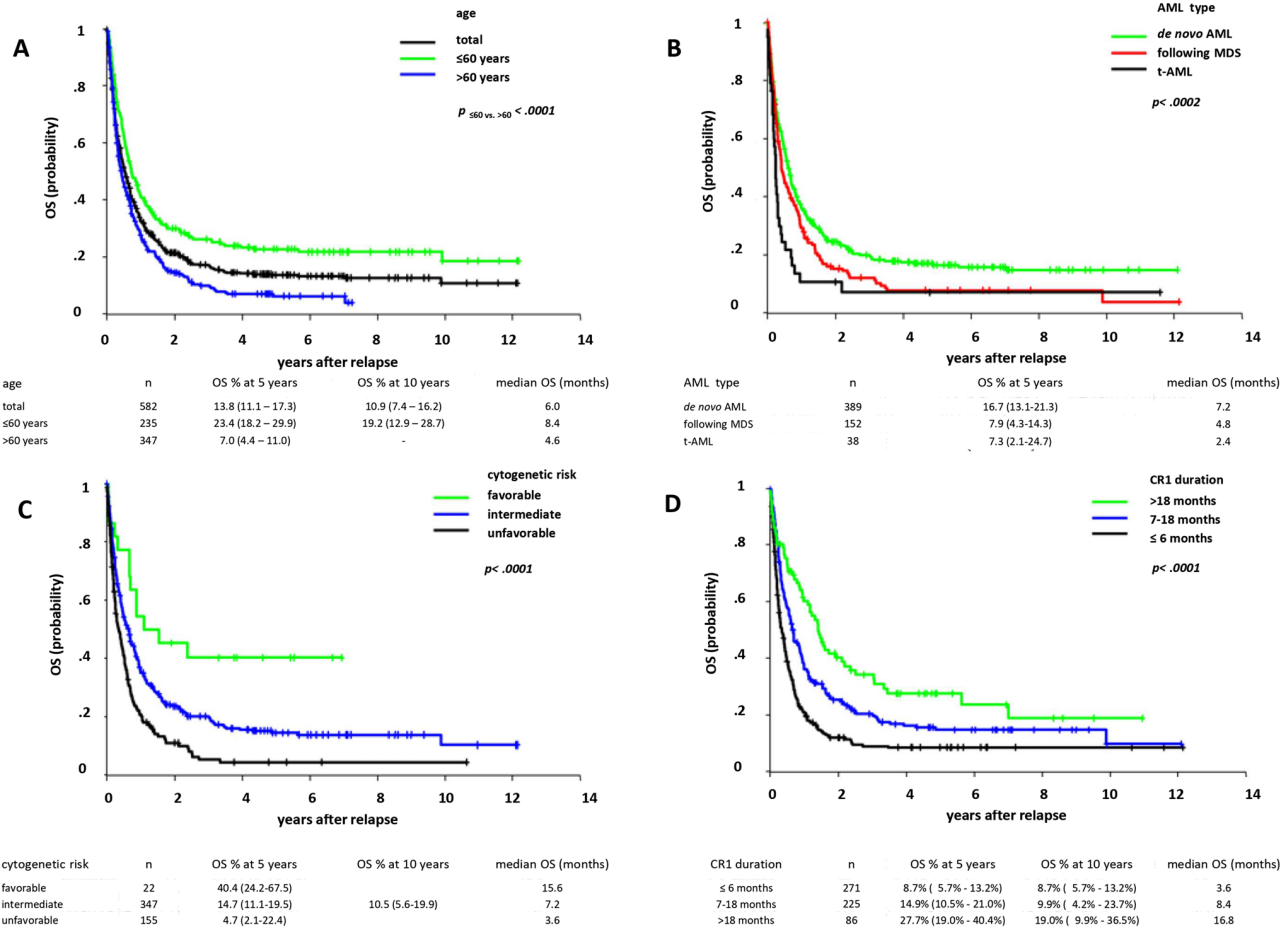
(Relapsed patients). **A** Overall survival (OS) of patients with AML after first relapse according to age. **B** Overall survival (OS) of patients with AML after first relapse according to de novo, secondary, and therapy-related AML. **C** Overall survival (OS) of patients with AML after first relapse according to favorable, intermediate, and unfavorable cytogenetics. **D** Overall survival (OS) of patients with AML after first relapse according to time interval CR1 and relapse in months

OS for relapsed patients was 10.9 (7.4–16.2) % at 10 years (Fig. 2A) and age decade dependent (suppl. Figure 2). A clear difference was noted between patients ≤ 60 and those > 60 years with OS rates of 23.4 (18.2–29.9) % and 7.0 (4.4–11.0) % at 5 years, respectively (Fig. 2A; p < 0.0001). OS was associated with AML type (Fig. 2B), cytogenetic risk (Fig. 2C), and duration of CR1 (Fig. 2D). The median OS in patients with monosomal karyotype was particularly low (2.4 versus 8.4 months; suppl. Figure 3; p < 0.0001) and similar to the median survival of relapsed patients transplanted in CR1 (median 3.6%; suppl. Figure 4; p < 0.01). *FLT3*-ITD and *NPM1* mutational status alone or in any of the possible combinations at initial diagnosis had no impact on OS after relapse (suppl. Figure 5).

**Fig. 3:**
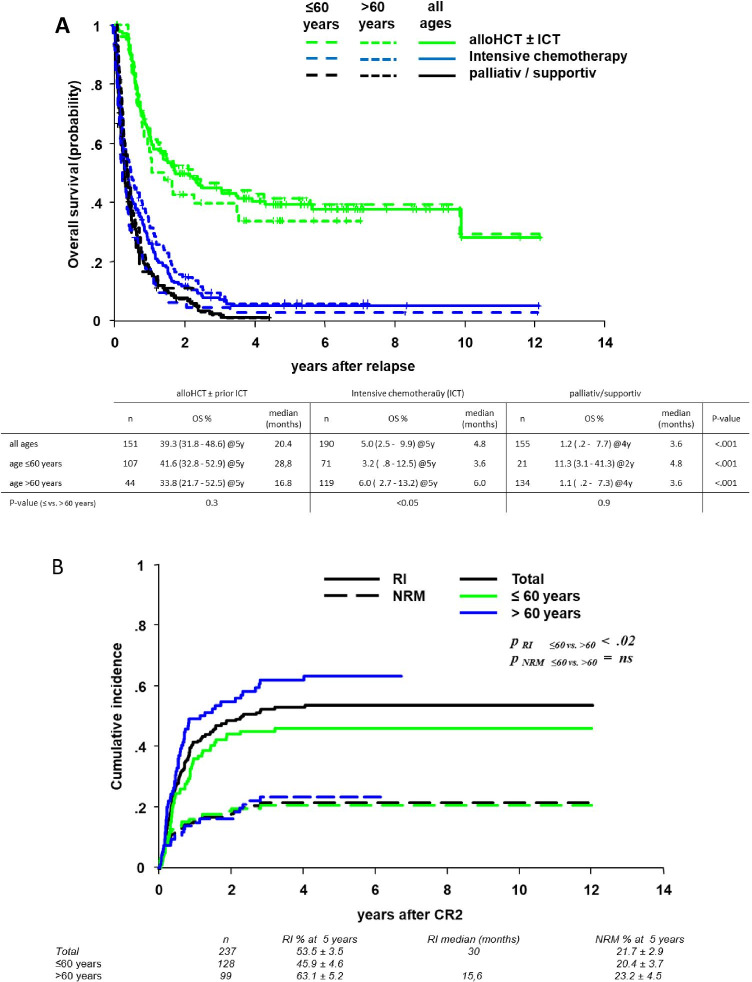
**A** Overall survival (OS) of patients with AML after first relapse according to age and therapy. Patients were treated with HCT ± induction chemotherapy (ICT), ICT alone, and palliative/supportive treatment. **B** Relapse incidence (RI) and non-relapse mortality (NRM) in patients with AML after first relapse (all ages, patients ≤ 60 years and > 60 years)

Patients with HCT ± ICT had an OS at 5 years of 39.3 (31.8–48.6) % (Fig. 3A) compared to 15.4 (6–39.9) % for those receiving DLI ± ICT/modified chemotherapy (suppl. Figure 6) and 5.0 (2.5–9.9) % for patients receiving ICT alone (p < 0.0001; Fig. 3A). The OS in patients with HCT ± ICT was durable up to 10 years and surprisingly not significantly different in younger as compared to elderly patients (Fig. 3A). The OS curves after palliative/supportive treatment did not show any age dependency (p = n.s.), while there was a difference in the 5-year OS (3% vs. 6%; p < 0.05) between younger and elderly patients with intensive chemotherapy.

Treatment with HCT and time interval CR1–relapse were age-independent variables for OS and in elderly patients, only monosomal vs. non-monosomal (Table 3). All other variables were interacting with age and not significant in the age-specific analysis. In the subgroup of patients with intensive chemotherapy, age, AML type, cytogenetics, and CR duration influenced survival (suppl. Figure 7), but cytogenetic risk, CR duration, and treatment with allogeneic HCT were the only independent risk factors, while all other characteristics interacted with age (suppl. Table 2). For palliative/supportive treatments, only cytogenetics and CR duration influenced OS (suppl. Figure 8), but only CR duration and non-monosomal vs. monosomal karyotype were independent risk factors (suppl. Table 2). The use of HMA agents in comparison to best supportive care was a beneficial factor in this treatment group, but not age or AML type. We finally were interested in analyzing the prognostic value of molecular marker in patients treated with intensive chemotherapy. As shown in suppl. Figure 9 (A–D), no significant differences in OS were observed between the different NPM1 and FLT3 combinations and the two age groups after intensive chemotherapy.

**Table 3:** Uni- and multivariate analysis for OS, LFS, RI, and NRM in patients with AML after first relapse (p-values or HR (95%CI))

		OS				LFS				RI				NRM				
Uni- or multivariate analysis		Uni	Multivariate			Uni	Multivariate			Uni	Multivariate			Uni		Multivariate		
		All p-value	All	≤ 60	Years	All p-value	All	≤ 60	Years	All p-value	All	≤ 60	Years	All p-value		All	≤ 60	Years
				> 60				> 60				> 60					> 60	
Age Continuous		< .001	^&^	n.s		< .01	^&^	n.s										
				n.s				n.s										
18–50/51–60/61–70/71–86 years		< .001				< .05				< .05	^&^	n.s./n.s		.08			^&^/ n.s	
Gender (female)		n.s				n.s				.06	.69 (.47–1.01)^§^	p < .01		n.s		n.s		
												n.s						
Type of AML De novo/following MDS/t-AML		< .001	^&^	n.s		.07^#^	^&#^			< .004^#^	^&^	n.s		n.s.^#^		n.s	n.s	
				n.s								< .01					p < .05	
Cytogenetic risk Favorable/intermediate/adverse		< .001	^&^	n.s		< .01^*^	^&*^	p < .05		n.s				n.s				
				n.s				n.s										
Monosomal/non-monosomal		< .001	^&^	n.s														
				2.25 (1.62–3.15)^§§§^														
NPM1 FLT3 FLT3/NPM1 FLT3/NPM1	wt vs. mut wt vs. *ITD* wt/wt vs. *ITD*/wt vs. wt/mut vs. *ITD*/mut wt/wt; wt/mut; *ITD*/mut vs. *ITD* /wt	n.s n.s n.s n.s	n.d			n.s n.s n.s n.s	n.d			n.s n.s n.s n.s	n.d			n.s n.s n.s n.s	n.d		n.d	
CR1–relapse time interval ≤ 6/7–18/ ≥ 18 months		< .001	.63 (.56–.72)^§§§^	P < .001		< .05	.72 (.58–.89)^§§^	n.s		< .05	.71 (.55–.93)^§§^	n.d		n.s				
				P < .001				p < .05										
Allogeneic HCT in CR1		< .01	^&^	n.s		n.s				n.s				n.s				
				^&^														
HCT in reinduction or consolidation		< .001	.40 (.31–.51)^§§§^	P < .001		< .01	.59 (.43–.81)^§§§^	n.s		< .01	.59 (.40–.86)^§§^	n.s		n.s			< .01	
				P < .001				n.s				n.s					n.s	

^&^Risk factors correlating with age; ^*^Favorable/intermediate vs. adverse cytogenetic risk; ^#^De novo vs. prior MDS + tAML; *n.d.*, not done; *n.s.*, not significant; ^§^p < .05; ^§§^p < .01; ^§§§^p < .001

LFS amounted to 24.9 (19.5–31.7) % at 5 years for all patients and 33.7 (26.2–43.5) % for younger patients (p = 0.008; suppl. Figure 10). There was no overall difference in LFS between patients according to NPM1 and FLT-ITD molecular markers (suppl. Figure 11). Similar to OS, LFS was influenced by age, cytogenetic risk, time interval CR–relapse, and HCT treatment. All but the last two variables were closely associated with age in the multivariate analysis (Table 3). For LFS after relapse, cytogenetic risk was an age-independent factor in younger and time interval in elderly patients.

A second relapse was the major complication in CR2 patients with 53.5 ± 3.5% at 5 years (Fig. 3B). RI amounted to 45.9 ± 4.6% at 5 years for younger and 63.1 ± 5.2% at 5 years for elderly patients was higher in unfavorable than in favorable cytogenetics and was influenced by age, type of AML (in elderly), gender (in younger), and type of treatment (Table 3). NRM for relapsed patients was 21.7 ± 2.9% (Fig. 3B) and influenced by HCT in younger and type of AML in elderly patients.

## Discussion

In contrast to untreated AML, for which cytogenetic and molecular prognostic factors are well established, prognostic factors for relapsed AML are less well defined. In the present study, we analyzed 582 patients with AML relapse out of 1621 patients covering the whole age spectrum of adult AML. Relapsed patients treated with allogeneic HCT had long-term OS of 39.3% at 5 years without significant differences between younger and older patients. DLI ± ICT and ICT alone had OS rates of ≤ 10% at 10 years. Clear differences were observed between younger and elderly patients in terms of disease characteristics (gender, AML type, cytogenetic risk, time interval from CR1 to relapse, and history of previous treatment), long-term outcome, and risk factors. Using multivariate analysis, we identified duration of first remission and allogeneic HCT as independent prognostic factors for OS. In contrast, mutational status of *FLT3-ITD* and *NPM1* at initial presentation had no significant impact on the prognosis after relapse. The major complication was age-dependent RI of 53.5% at 5 years. NRM was age independent and resulted in 21.7% at 5 years.

Our study of a large number of patients covering the AML-typical age spectrum over a long observation period has several implications. First, it confirms the high RI of patients with newly diagnosed AML in CR1 of 53.1% rising to 63.5% at 5 years in patients > 60 years. Strategies to reduce RI are urgently needed. Maintenance approaches based on conventional chemotherapy, immunotherapy, HMA, and targeted small molecules have been explored. No data so far have been convincing enough to establish one approach as the standard of care, although recent trials in AML subgroups with targeted therapy are promising [18]. The more frequent use of the most potent antileukemic approach [19], allogeneic HCT, in high risk and intermediate risk AML patients in CR1 may be an option in the light of the continuous reduction in transplant-related mortality [20]. The use of HCT should be increased not only in the younger but also in the elderly population taking advantage of new low-toxicity technologies and the availability of donors for almost every patient [21, 22]. Indications for HCT have been described previously [20] and take into account the risk of HCT, the comorbidity of the patients, and the relapse incidence but should also consider the outcome of relapsed patients. Based on these considerations, at least 60% of patients with AML in CR1 may need an HCT. In our cohort, the HCT rate was 37.3% in all patients with CR1, 46.6% for patients ≤ 60 years, and 27.6% for > 60 years. A broader indication in younger patients with AML in CR1 and even more so in elderly patients may be aspired. In addition, MRD-guided therapy might improve the results. Finally, the identification of driver mutations and the availability of targeted small molecules for maintenance may help to reduce the relapse rate in CR1 [23].

Obtaining CR2 after relapse of AML is of fundamental importance for long-term outcome. Numerous salvage regimens have been used and some have been compared in prospective trials [3, 24-27]. CR rates are roughly 50% with many of the protocols, but CR duration is rather short and median OS only about 6 months. Even a liposomal formulation of cytarabine and daunorubicin did not show a survival advantage in refractory AML except in a smaller subgroup [28]. It is not expected that chemotherapies or combination of chemotherapies will improve these results. Less toxic, targeted therapies to driver mutations might be more effective in inducing CR in selective AML subpopulations, as in the case of *FLT3-ITD* [29, 30] or IDH 1 or 2 inhibitors in patients carrying respective mutations [31]. Therapies with HMA with or without anti-apoptotic pathways inhibitors might be an option in patients with contraindication for intensive chemotherapy or even in relapsed AML first line [32-34].

Long-term OS data of our study highlight the key role of HCT in the treatment of relapsed AML not only in younger but also for the first time in elderly patients. Without HCT, OS amounts to only ≤ 10% at 10 years. Similar results were reported by the ECOG‐ACRIN Cancer Research Group describing a 5‐year OS of only 10% in younger patients [35]. Results from trials performed between 1983 and 1997 in patients ≤ 55 years report an OS of 9% in comparison to the 23.4% OS at 5 years in our analysis [7]. For elderly patients, OS at 5 years was reported to be 6% compared to the 7% seen in our study. Although HCT has been described as beneficial mainly in younger patients, our results suggest that allogeneic HCT in CR2 is the treatment with the highest long-term OS (37.9%) at 5 years and that elderly patients have results comparable to younger patients. Similar to our previously described concept of early HCT after achieving CR in high risk patients, performing HCT in CR as early and in as many patients as possible may further improve results in CR2 [36]. Results of DLI ± intensive/modified chemotherapy seem not to be an alternative to HCT.

Risk factors for CR and outcome in first relapse have been identified previously on smaller and younger populations [3]. Keating et al. demonstrated that age is an important predictor for response and survival [8] and Estey et al. that duration of first remission is an important predictor for survival [4]. Breems et al. confirmed four important prognostic indicators for survival: cytogenetics at initial diagnosis [t(16;16) or inv(16) being favorable], age at relapse, duration of first CR, and allogeneic HCT before relapse (unfavorable) [37]. In a smaller study with 81 relapsed and 57 refractory younger patients (median age 55 years), CR duration < 12 months, *FLT3-ITD*-positive status, and high-risk cytogenetics emerged as the three strongest independent adverse prognostic factors for OS and event-free survival [38]. In a study of the Spanish PETHEMA group, high-risk cytogenetics and t(8;21) at diagnosis, no previous allogeneic HCT and relapse-free interval < 12 months were associated with lower CR/CRi (median age 54 years). Of note, previous allogeneic HCT was a favorable prognostic factor in the PETHEMA study in contrast to other studies [39]. The largest study to date analyzed 1307 AML relapses out of 2170 patients in CR1 (60.2%) [40] according to curative (median age 53.6 years) and palliative treatment (median age 60.5 years). CR was observed in 38.4% of patients, with CR duration > 18 months, biallelic *CEPBA* mutation, and core binding factor-AML being favorable, while adverse cytogenetics and *FLT3-ITD* were negative prognostic factors for achieving CR or CRi. Interestingly, neither age, previous treatment with HCT, nor *NPM1* mut were associated with response to salvage therapy. These results can only be compared to our younger patient population in which no impact of *FLT3-ITD* at diagnosis was found.

The current study has strengths and limitations. The prospective inclusion of all AML patients from diagnosis (with corresponding AML-typical age distribution and median age of 62 years) to relapse and all possible therapies of relapse are definitely strengths. Furthermore, a high proportion of patients over the age of 60 years was treated in a curative attempt at diagnosis (> 67%) and at relapse (53.8%). Limitations include lack of information on cytogenetic and molecular alterations at the time of relapse, in part also at initial diagnosis, lack of information on allelic ratios of FLT3 mutations, and missing ECOG and comorbidity indices impacting clinical outcome. However, entry of consecutive patients from diagnosis and the AML-typical age distribution may argue against biases. Furthermore, the use of non-myeloablative, less toxic conditioning regimen (Fludarabin/200 cGy total body irradiation, cyclosporine, and mycophenolate mofetil) and unrelated donors in elderly patients without outcome differences between 60–64, 65–69, and > 70 years may have played an important role [41].

Our study contributes to the knowledge and outcome on relapsed AML. While results in relapsing patients remain poor overall, results in subgroup of patients have shown clear improvement. Our data support previous studies showing increasing age, a shorter CR duration, and type of AML to be the strongest prognostic factors for CR2 and CR2 an important determinant for HCT. It is expected that with the use of targeted therapy and/or with use of HMA in combination with Bcl-2 inhibitors, CR rates will increase and may improve the results of HCT, if deeper CR rates are achieved. Currently, long-term results can only be obtained if CR2 is followed by HCT, which in our study was used in 84.4% of younger and in 47.4% of elderly patients in CR2. Explicitly, 53.8% (n = 163) of elderly patients received ICT, but only 44 patients received HCT. Increasing the rate of allogeneic HCT is no doubt the most interesting approach.

An accurate molecular analysis is required at the time of relapse to identify patients with driver mutations for whom targeted therapy is feasible and to facilitate subsequent MRD monitoring. Considering all the risk factors and heterogeneity of the disease, it is unrealistic to expect an improvement in OS across all AML patients. Stratification and the use of approaches tailored to individual subgroups will clearly be necessary. In this respect, identification of *FLT3-ITD* patients and targeted treatment with potent TKI inhibition like quizartinib and gilteritinib, monitoring MRD, and the use of HCT represent the most promising approach. Increasing CR2 rates, the use of HCT especially in elderly and reducing relapses will be the way to go.

## Supplementary Information

Below is the link to the electronic supplementary material.

Supplementary file1 (DOCX 1992 KB)
