# Supplementary material for: Allogeneic hematopoietic stem cell transplantation improves long-term outcome for relapsed AML patients across all ages: results from two East German Study Group Hematology and Oncology (OSHO) trials
Source: Ann Hematol. 2021 Jul 7;100(9):2387–98. doi: 10.1007/s00277-021-04565-1 (PMC8357692; doi:10.1007/s00277-021-04565-1)
Supplement: Supplementary file 1 — Supplementary file1 (DOCX 1992 KB) [file 277_2021_4565_MOESM1_ESM.docx]

**Suppl. Table 1: Risk factors for achieving CR2 in patients with AML in first relapse (uni- and multivariate analyses)**

|  | **CR2**  **all patients** | | **CR2**  **≤60 years** | **CR2**  **>60 years** |
| --- | --- | --- | --- | --- |
|  | **uni-**  **variate** | **multi-**  **variate** | **multi-**  **variate** | **multi-**  **variate** |
| **age**  continuous  18 - 50 / 51 - 60 / 61 - 70 / 71 - 86 years | ***< .0001***  ***< .0001*** | ******* | ***< .05*** | ******* |
| **gender** | n.s. |  | n.s. | n.s. |
| **type of AML** *de novo / prior MDS / t-AML* | ***< .001*** | *** | n.s. | n.s. |
| **Cytogenetic risk** *favorable/intermediate/adverse*  monosomal/non-monosomal | ***< .01***  ***< .0001*** | *****  ******* | ***< .05*** | ***< .05*** |
| **NPM1** wt/mut  **FLT3** wt/ITD  **FLT3/NPM1** wt/wt vs. ITD/wt vs. wt/mut vs. ITD/mut  **FLT3/NPM1** wt/wt; wt/mut; ITD/mut vs. ITD/wt | n.s.  n.s.  n.s.  n.s. | n.d. | n.d. | n.d. |
| **intervall CR -> relapse** *≤ 6 / 7 - 18 / ≥ 18 months* | ***< .0001*** | ***< .0001*** | ***< .01*** | n.s. |
| **alloSCT in CR1** | ***< .05*** | ******* | ***< .01*** | n.s. |
| **Treatment** ICT vs. HCT vs. DLI vs. palliative vs. BSC | ***< .0001*** | ***< .0001*** |  | ***< .0001*** |

^* significant interaction between age and AML subtype, cytogenetic risk, HCT in CR1^

|  | **OS**  **intensive chemotherapy**  **n=368** | | **OS**  **palliative/supportive therapy**  **(n=155)** | |
| --- | --- | --- | --- | --- |
|  | **univariate** | **multivariate** | **univariate** | **multivariate** |
| **age**  continuous  18 - 50 / 51 - 60 / 61 - 70 / 71 - 86 years | ***<.01***  ***<.05*** | ******** | n.s.  n.s. |  |
| **gender** | n.s. |  | n.s. |  |
| **type of AML** *de novo / prior MDS / t-AML* | ***< .05*** | ****** | n.s. |  |
| **Cytogenetic risk** *favorable/intermediate/adverse*  monosomal/non-monosomal | ***<.01***  ***<.01*** | ***<.05***  *n.s.* | ***<.05***  ***< .0001*** | **n.s.**  ***<.001*** |
| **NPM1** wt vs. mut  **FLT3** wt vs.ITD  **FLT3/NPM1** wt/wt vs. ITD/wt vs. wt/mut vs. ITD/mut  **FLT3/NPM1** wt/wt; wt/mut; ITD/mut vs. ITD/wt | n.s.  n.s.  n.s.  n.s. |  | n.s.  n.s.  n.s.  n.s. |  |
| **CR - Relapse time interval** *≤ 6 / 7 - 18 / ≥ 18 months* | ***< .0001*** | ***< .0001*** | ***<.05*** | ***<.05*** |
| **allogeneic HCT in CR1** | ***<.05*** | ******** | n.s. |  |
| **allogeneic HCT as consolidation in CR2**  **palliative/supportive**  **HMA vs. low dose chemotherapy/palliative** | ***< .0001*** | **< .0001** | n.d.  ***< .0001***  ***< .0001*** | n.d.  n.s.  ***< .0001*** |

**Suppl. Table 2: Uni- and multivariate analysis for OS after relapsed AML according to treatment**

** significant interaction between age and AML subtype and HCT In CR1. Intensive treatment (intensive chemotherapy ± hematopoietic cell transplantation; donor lymphocyte infusion with chemotherapy); BSC, best supportive care; HMA, hypomethylating agents

**Suppl. Figure 1:** Relapse Free Survival according to *FLT3* status in patients ≤ 60 years

**0**

**2**

**4**

**6**

**8**

**10**

**12**

**14**

**years after CR/CRi**

**0**

**.2**

**.4**

**.6**

**.8**

**1**

**Relapse-free survival (probability)**

**FLT3 wt**

**FLT3-ITD**

***p < .02***

|  | *n* | *RFS % at 10 years* | *median (months)* | |
| --- | --- | --- | --- | --- |
| *FLT3 wt* | *324* | *45.9 (40.0 - 52.6)* | *57.6* |  |
| *FLT3 ITD* | *92* | *31.1 (22.0 - 43.9)* | *15.6* |  |

**Suppl. Figure 2:** OS of AML patients according to age decades (18 – 50; 51 – 60; 61 – 70; 71 – 86 years)

**0**

**2**

**4**

**6**

**8**

**10**

**12**

**14**

**years after first relapse**

**0**

**.2**

**.4**

**.6**

**.8**

**1**

**OS (probability)**

**18 - 50 years**

**51 - 60 years**

**61 - 70 years**

**71 - 86 years**

***p < .0001***

| Age (years) | n | OS % at 5 years | median (months) |
| --- | --- | --- | --- |
| 18 - 50 | 128 | 25.1 (18.3 - 34.4) | 10.8 |
| 51 - 60 | 118 | 21.8 (15.0 - 31.7) | 6.0 |
| 60 - 70 | 213 | 7.5 ( 4.4 - 13.0) | 4.8 |
| 71 - 86 | 123 | 2.4 ( 2.1 - 12.6) | 4.8 |

**Suppl. Figure 3:** OS according to monosomal vs. non-monosomal

**0**

**2**

**4**

**6**

**8**

**10**

**12**

**14**

**years after first relapse**

**0**

**.2**

**.4**

**.6**

**.8**

**1**

**OS (probability)**

**non-monosomal**

**monosomal**

***p < .0001***

| cytogenetics | n | OS % at 5 years | median (months) |
| --- | --- | --- | --- |
| non-monosomal | 448 | 15.4 (12.0 - 19.8) | 8.4 |
| monosomal | 76 | 2.2 ( 0.3 - 14.2) | 2.4 |

**Suppl. Figure 4:** OS according to allogeneic HCT in CR1

**0**

**2**

**4**

**6**

**8**

**10**

**12**

**14**

**years after first relapse**

**0**

**.2**

**.4**

**.6**

**.8**

**1**

**OS (probability)**

**no allogeneic HCT in CR1**

**allogeneic HCT in CR1**

***p = .003***

|  | *n* | *OS % at 5 years* | *median OS (months)* |
| --- | --- | --- | --- |
| no allogeneic HCT in CR1 | *454* | *15.3 (12.1 - 19.4)* | *7.2* |
| allogeneic HCT in CR1 | *128* | *9.0 ( 4.9 - 16.6)* | *3.6* |

**Suppl. Figure 5:** OS according to FLT3/NPM1 mutation status

**0**

**2**

**4**

**6**

**8**

**10**

**12**

**14**

**years after first relapse**

**0**

**.2**

**.4**

**.6**

**.8**

**1**

**OS (probability)**

***p = n.s.***

**FLT3 / NPM1-wt**

**FLT3-ITD / NPM1-wt**

**FLT3 / NPM1-mut**

**FLT3-ITD / NPM1-mut**

| **FLT3 / NPM1** | **n** | **OS % at 5 years** | **median (months)** |
| --- | --- | --- | --- |
| **wt / wt** | **233** | **11.1 ( 7.5 - 16.6)** | **6.0** |
| ***ITD* / wt** | **52** | **18.4 ( 9.3 - 36.2)** | **7.2** |
| **wt / mut** | **89** | **16.7 ( 9.9 - 28.0)** | **6.0** |
| ***ITD* / mut** | **36** | **23.5 (12.9 - 43.2)** | **4.8** |

**Suppl. Figure 6:** OS according to treatment (allogeneic HCT; ICT, intensive chemotherapy; DLI, Donor Lymphocyte Infusion; HMA, hypomethylating agents; mod. CT, modified non-intensive chemotherapy)


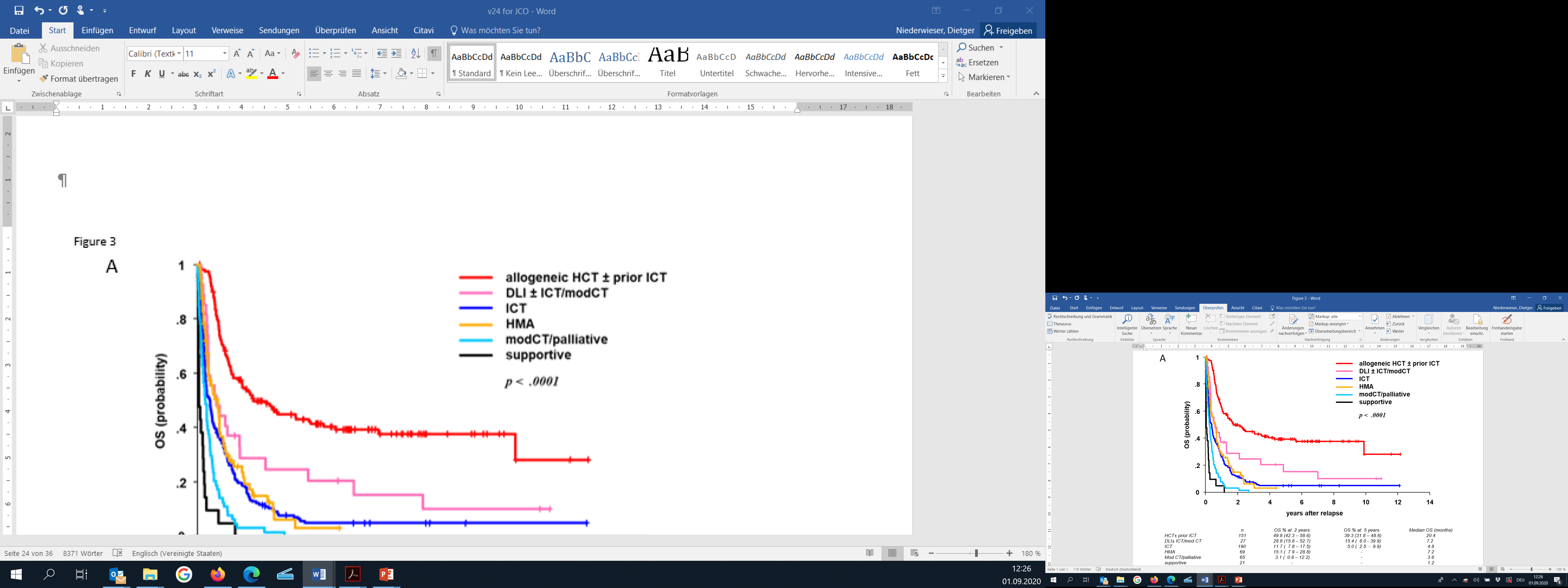


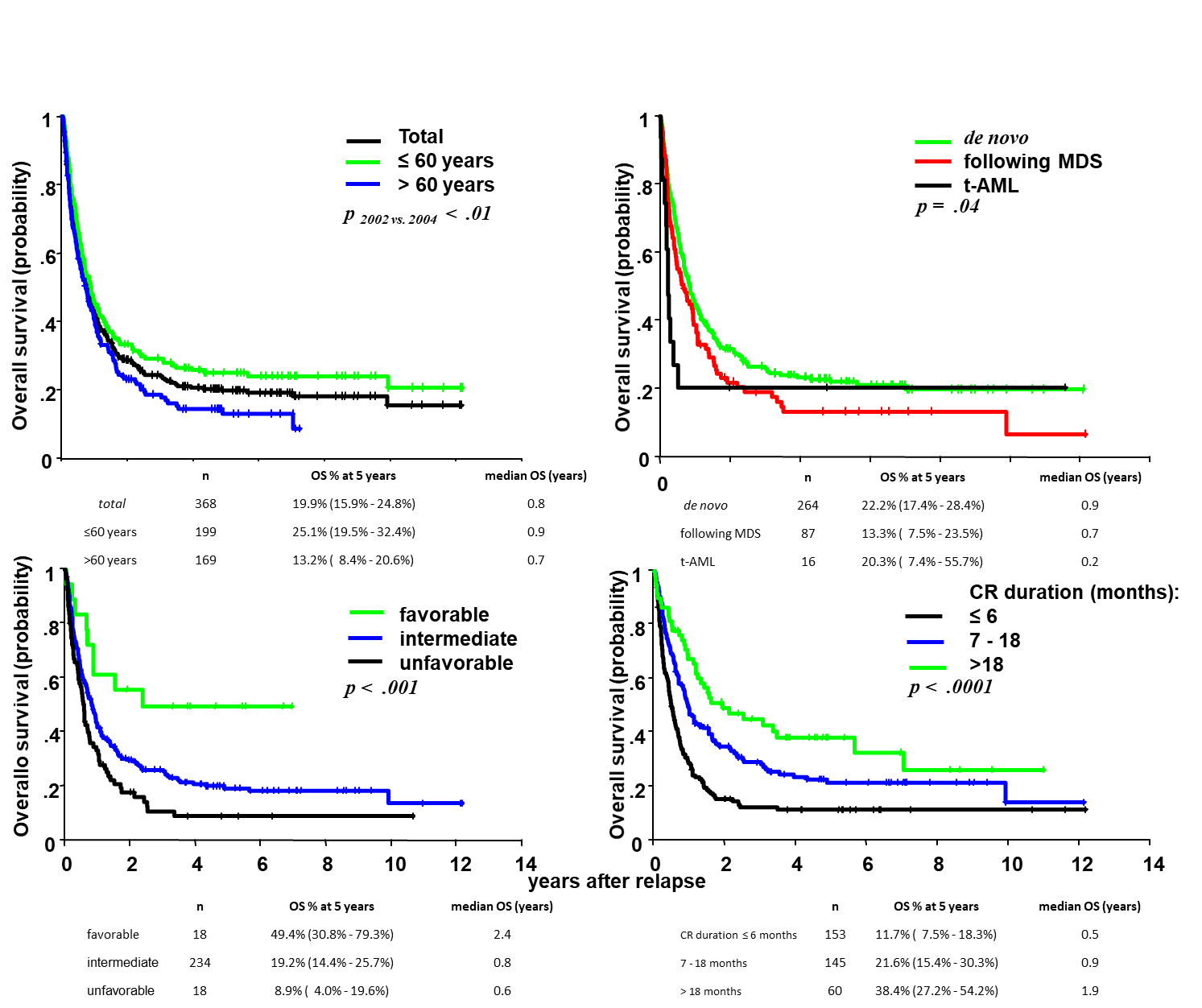


**Suppl. Figure 7:** OS in patients with intensive chemotherapy according to age, AML type, cytogenetic risk and CR – relapse interval


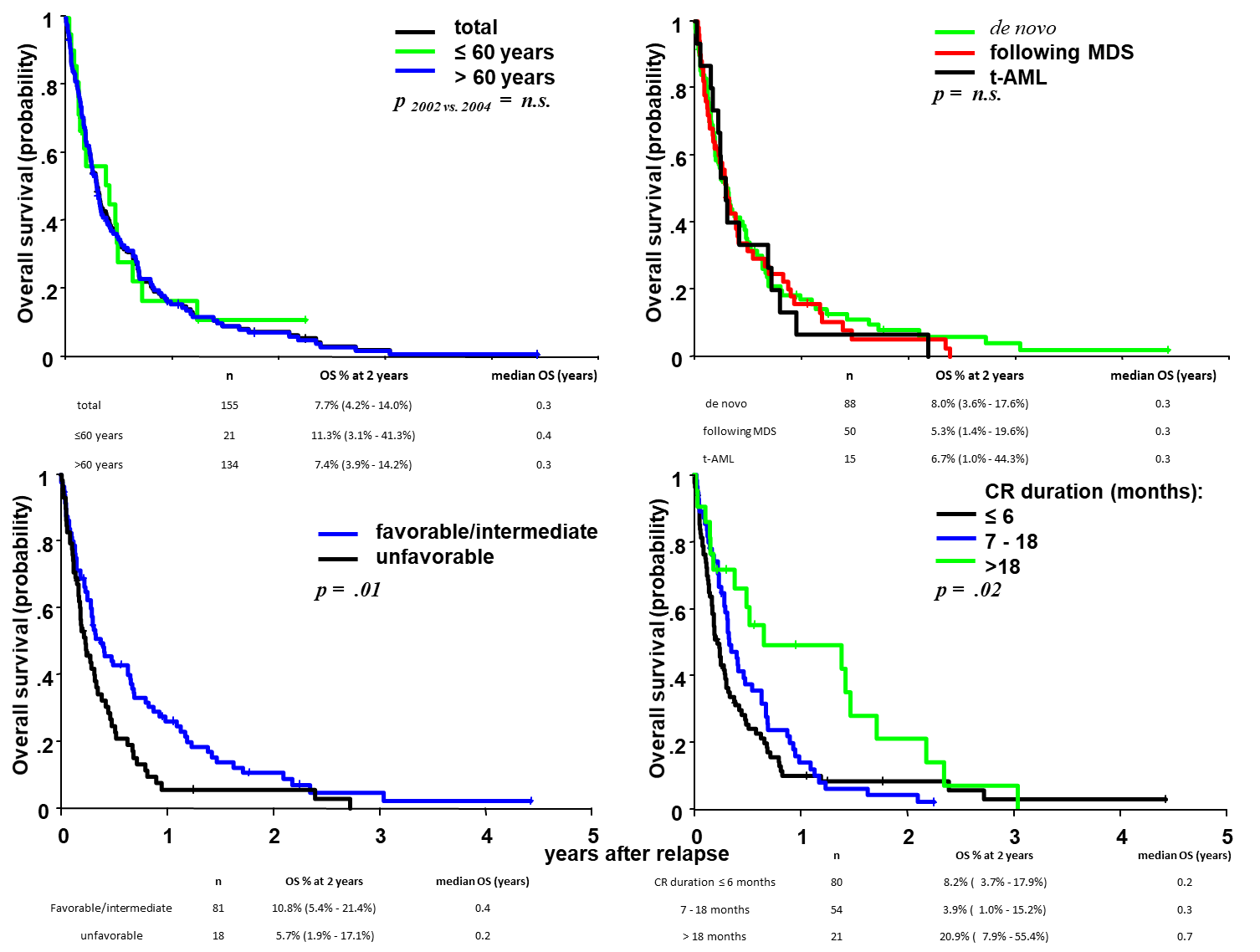


**Suppl. Figure 8:** OS in patients with palliative/supportive treatment according to age, AML type, cytogenetic risk and CR – relapse interval

**
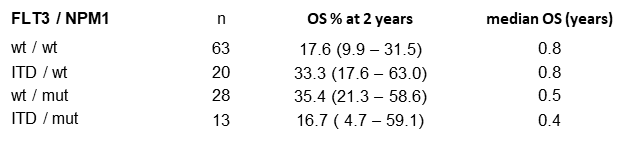

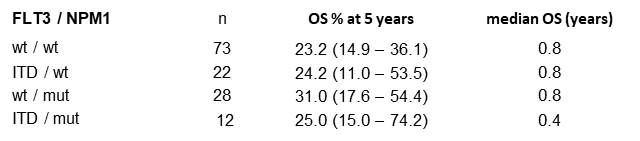

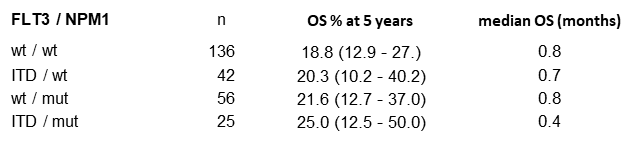

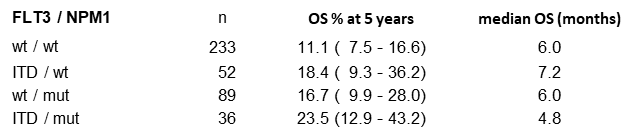

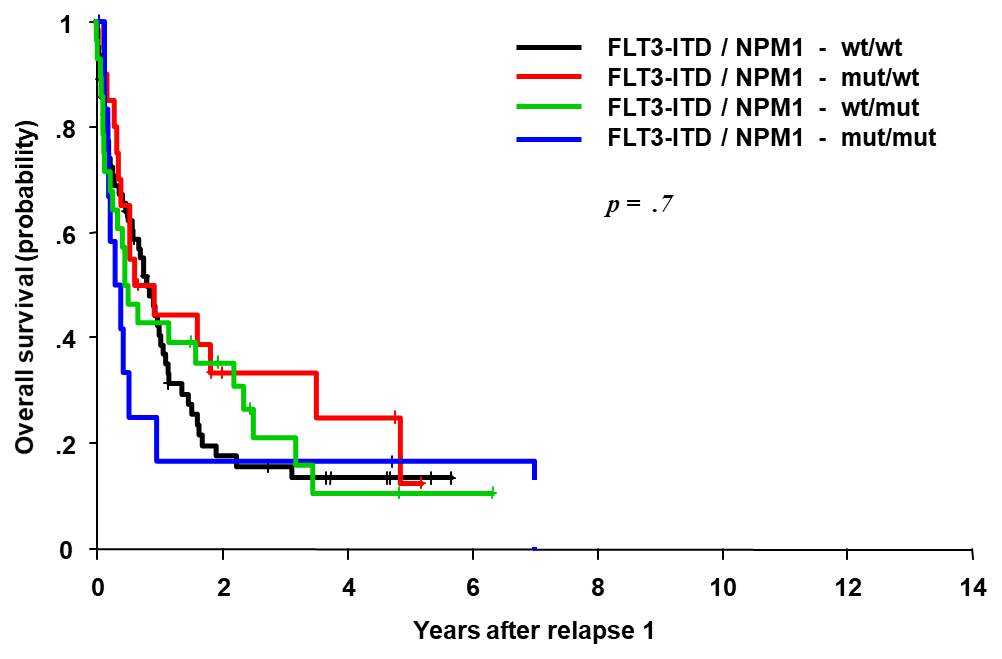

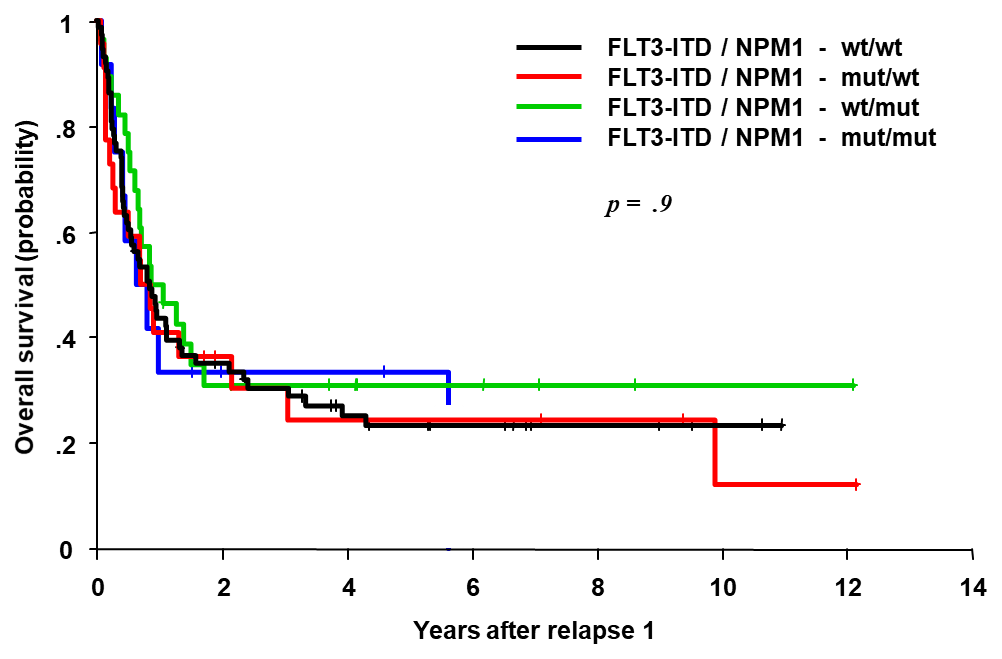

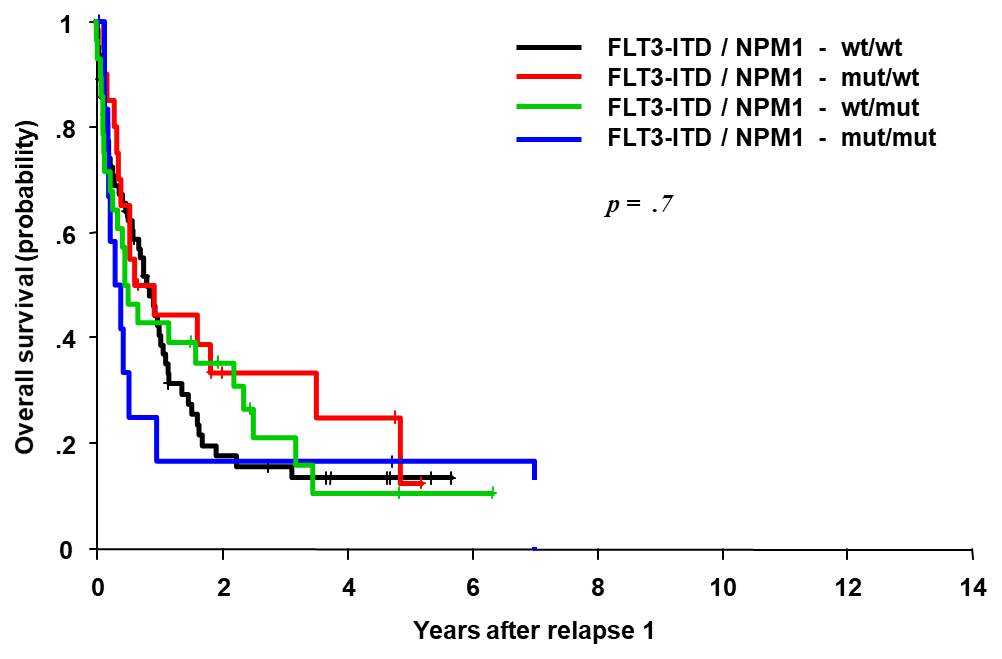

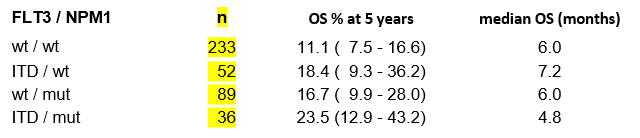

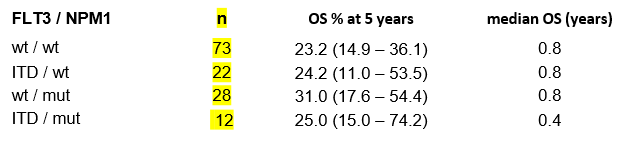

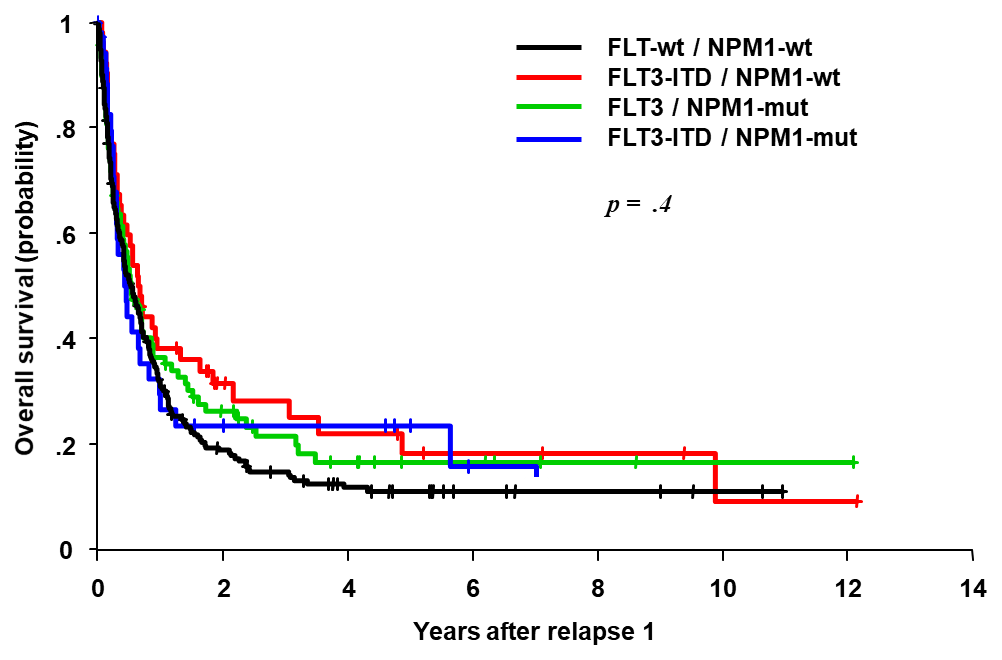
**

OS intensive chemotherapy all ages (n=259)

OS intensive chemotherapy >60 years(n=124)

OS intensive chemotherapy <60 years (n=135)

**FLT3 / NPM1-wt**

**FLT3-ITD / NPM1-wt**

**FLT3 / NPM1-mut**

**FLT3-ITD / NPM1-mut**

p = n.s.

**Suppl. Figure 9:** OS after intensive chemotherapy according to molecular risk factors and age

D

C

B

A

years after relapse

p = n.s.

p = n.s.

p = n.s.

**0**

**2**

**4**

**6**

**8**

**10**

**12**

**14**

**Years after relapse 1**

**0**

**.2**

**.4**

**.6**

**.8**

**1**

***p = .7***

OS all treatments (n= 410)

**0**

**2**

**4**

**6**

**8**

**10**

**12**

**14**

**years after CR2**

**0**

**.2**

**.4**

**.6**

**.8**

**1**

**LFS (probability)**

**Total**

**≤ 60 years**

**> 60 years**

***p _≤ 60_* *_vs. >60_ = .008***

**Suppl. Figure 10:** LFS of patients with relapsed AML according to age

|  | *n* | *LFS % at 5 years* | *median LFS (months)* |
| --- | --- | --- | --- |
| *total* | *227* | *24.9 (19.5-31.7)* | *9.6* |
| AML02, ≤60 years | *128* | *33.7 (26.2-43.5)* | *10.8* |
| AML04, >60 years | *99* | *13.8 (8.1-23.4)* | *7.2* |

***p = .5***

**0**

**2**

**4**

**6**

**8**

**10**

**12**

**14**

**Years after relapse 1**

**0**

**.2**

**.4**

**.6**

**.8**

**1**

**Leukemia free survival (probability)**

**Suppl. Figure 11:** LFS of patients with relapsed AML according to molecular marker

**FLT3 / NPM1-wt**

**FLT3-ITD / NPM1-wt**

**FLT3 / NPM1-mut**

**FLT3-ITD / NPM1-mut**

| **FLT3 / NPM1** | **n** | **OS % at 5 years** | **median (months)** |
| --- | --- | --- | --- |
| **wt / wt** | **82** | **20.0% (12.7 - 31.5)** | **8.4** |
| **ITD / wt** | **25** | **20.3% ( 8.8 - 46.8)** | **9.6** |
| **wt / mut** | **36** | **30.3% (17.6 - 52.0)** | **14.4** |
| **ITD / mut** | **11** |  | **13.2** |

FLT3-ITD / NPM1 = wt / wt 20.0% (12.7 - 31.5) @5y, median 0.7y

FLT3-ITD / MPN1 = mut / wt 20.3% ( 8.8 - 46.8) @5y, median 0.8y

FLT3-ITD / MPN1 = wt / mut 30.3% (17.6 - 52.0) @5y, median 1.2y

FLT3-ITD / MPN1 = mut / mut median 1.1y
